# Possible inhibition of GM-CSF production by SARS-CoV-2 spike-based vaccines

**DOI:** 10.1186/s10020-021-00313-3

**Published:** 2021-05-22

**Authors:** Jianhua Li, Ping Wang, Kevin J. Tracey, Haichao Wang

**Affiliations:** 1 The Feinstein Institutes for Medical Research, Northwell Health, 350 Community Drive, Manhasset, NY 11030 USA; 2 Donald and Barbara Zucker School of Medicine at Hofstra/Northwell, 500 Hofstra Blvd, Hempstead, NY 11549 USA

**Keywords:** GM-CSF, SARS-CoV-2, Spike protein, Antibody

## Abstract

A SARS-like coronavirus 2 (SARS-CoV-2) has caused a pandemic Coronavirus Disease 2019 (COVID-19) that killed more than 3.3 million people worldwide. Like the SARS-CoV, SARS-CoV-2 also employs a receptor-binding motif (RBM) of its spike protein to bind a host receptor, the angiotensin-converting enzyme 2 (ACE2), to gain entry. Currently, several mRNA or adenoviral vaccines encoding for the spike protein of SARS-CoV-2 are being used to boost antibodies capable of inhibiting spike-ACE2 interaction and viral entry. However, recent evidence has also suggested an anti-inflammatory effect of spike-reactive antibodies, suggesting that some SARS-CoV-2 spike-based vaccines may elicit protective antibodies capable of inhibiting GM-CSF production and COVID-19 progression.

## Background

The recent emergence and rapid spread of SARS-like coronavirus 2, SARS-CoV-2, has caused a pandemic COVID-19 that is catastrophically harming human health (Qiang et al. 2021). As of 12 May 2021, approximately 159 million people have been infected, leading to more than 3,300,000 deaths worldwide (https://www.who.int/emergencies/diseases/novel-coronavirus-2019). Like the β-coronavirus that caused the 2003 outbreak of the severe acute respiratory syndrome (SARS-CoV), SARS-CoV-2 virus also employs its envelope spike (S) glycoproteins to bind a host cell surface receptor, the angiotensin-converting enzyme 2 (ACE2), to gain host cell membrane fusion and viral entry (Hoffmann et al. 2020).

### SARS-CoV-2 Spike-reactive antibodies impair viral entry

To boost adaptive antibody responses against SARS-CoV-2 infections, several mRNA and adenoviral vaccines encoding a surface fragment of a SARS-CoV-2, the spike (S) protein, are currently being employed worldwide to fight against COVID-19. It is hoped that antibodies raised against SARS-CoV-2 S protein may inhibit viral interaction with host ACE2 receptor, thereby preventing viral entry (Amanat and Krammer 2020). Indeed, neutralizing antibodies targeting the receptor-binding domain (RBD) or the receptor-binding motif (RBM) of SARS-CoV-2 S protein were found in the blood of COVID-19 patients (Zost et al. 2020; Pinto et al. 2020), and some of which could indeed impair viral entry (Zost et al. 2020; Pinto et al. 2020).

### SARS-CoV-2-reactive antibodies specifically inhibit GM-CSF production

Recent evidence suggested that ACE2 might also be expressed in human peripheral blood mononuclear cells (PBMCs) (Zhang et al. 2020; Qiang et al. 2021), which could produce pro-inflammatory cytokines [e.g., tumor necrosis factor (TNF), interleukin-1β (IL-1β) and IL-6) and chemokines [e.g., IL-8 and macrophage inflammatory protein-1β (MIP-1β)] in response to SARS-CoV-2 S protein stimulation (Qiang et al. 2021). However, it was previously unknown how SARS-CoV-2 spike protein-binding monoclonal antibodies (mAbs) affect the SARS-CoV-2-elicited innate immune responses. Accordingly, we have recently generated recombinant protein corresponding to the receptor binding domain (RBD, residue 319–541) and receptor binding motif (RBM, residue 437–508) of SARS-CoV-2 spike (S) protein, and employed them to assess the ACE2-binding properties and screen for RBM-binding mAbs using Surface Plasma Resonance (SPR) technique (Qiang et al. 2021).

Although expressing recombinant proteins in *E. coli* is relatively easy and cost-effective, cysteine-rich proteins (such as the full-length spike protein of SARS-CoV-2) can be difficult to produce in prokaryotes partly because the reducing environment of the bacterial cytoplasm favors the formation of incorrect disulfide bond and production of insoluble protein aggregates (i.e., inclusion bodies) that are often difficult to refold correctly after denaturation. Indeed, even a small fragment of the SARS-CoV-2 spike protein (such as the RBM and RBD) formed insoluble inclusion bodies *in E. coli,* and a high concentration of denaturant (8 M urea) had to be employed to solubilize these inclusion bodies before subsequent chromatography purification and refolding (Qiang et al. 2021). To prevent excessive oxidation and cross‐linking of the nine and two cysteines in RBD and RBM, respectively, these recombinant proteins were renatured and refolded in a buffer containing a reducing agent [Tris (2‐carboxyethyl) phosphine (TCEP)]. Even so, recombinant RBD still exhibited an extremely low affinity to human ACE2 with an estimated K_D_ of 161,000 nM, possibly because the cysteine-rich RBD was not refolded into a “correct” conformation suitable for RBM‐ACE2 interaction, as the high probability of “incorrect” disulfide cross-linking was factorially proportional to the number of cysteine residues (Qiang et al., 2021).

In contrast, SPR analysis revealed an equilibrium binding constant (K_D_) of 42.5–64.1 nM for ACE2-RBM binding (Qiang et al. 2021), which was approximate to previously reported K_D_ (15–44.2 nM) for SARS-CoV-2 spike-ACE2 interactions (Wrapp et al. 2020). By conjugating recombinant RBM on a sensor chip, we found two monoclonal antibodies that dose-dependently interacted with SARS-CoV-2 RBM, with an estimated K_D_ of 17.4 and 62.8 nM, respectively (Qiang et al. 2021). These estimated K_D_ were comparable to that of other SARS-CoV-2 RBD-binding neutralizing antibodies (K_D_ = 14–17 nM) derived from COVID-19 patients (Rogers et al. 2020). These RBM-reactive mAbs competitively inhibited RBM-ACE2 interactions in vitro (Qiang et al. 2021), and selectively impaired the RBM-induced secretion of the granulocyte macrophage colony-stimulating factor (GM-CSF) without affecting the release of other cytokines (e.g., IL-1β, IL-6, IL-10 and TNF) or chemokines [MIP-1δ and monocyte chemoattractant protein-1 (MCP-1)] by human PBMCs (Qiang et al. 2021).

The intricate mechanism by which RBM-reactive antibodies selectively blocked the RBM-induced GM-CSF induction without affecting the release of other cytokines/chemokines remains an exciting subject of future investigation. Because human or murine GM-CSF does not contain any segment that even remotely resembles the epitope sequence (NDALYEYLRQ) of the TN/RBM-reactive antibodies, these antibodies are not expected to bind human or murine GM-CSF or to interfere with its immune-detection by Cytokine Antibody Arrays. Furthermore, our immunoblotting analysis revealed that these TN/RBM-reactive antibodies did not cross-react with any proteins in the whole-cell lysates of murine macrophages or human peripheral blood mononuclear cells (huPBMCs), confirming a lack of cross-reactivity of these TN/RBM-reactive mAbs to any other endogenous proteins of innate immune cells (Qiang et al. 2021). We speculate that SARS-CoV-2 spike protein activates innate immune cells through multiple signaling molecules that might include ACE2 and other yet-to-be identified pathogen pattern recognition receptors (PRRs). It is thus possible that some RBM-reactive antibodies might selectively prevent its interaction with a receptor involved in the GM-CSF induction, but did not interfere with its engagement with other pattern recognition receptors responsible for the induction of other cytokines or chemokines. Furthermore, we did not generate full-length spike protein *in E. coli* to confirm its innate immune stimulatory properties, because the full-length spike protein might similarly fail to fold correctly after urea denaturation. Therefore, future studies are needed to confirm the innate immune stimulatory properties of the full-length spike protein of SARS-COV-2 using recombinant proteins expressed in other eukaryotic cells. Nevertheless, our surprising findings fully supported the emerging notion that GM-CSF might be a key biomarker for SARS-CoV-2-induced cytokine storm in a subset of COVID-19 patients with more severe pneumonia often escalating to respiratory failure and death (Hue et al. 2020; Gibellini et al. 2020; Blot et al. 2020; Thwaites et al. 2021; Zhao et al. 2021).

### GM-CSF as a biomarker of COVID-19

While comparing the immunopathology of acute respiratory distress syndrome (ARDS) between patients with COVID-19 and non-COVID-19, Hue S et al. found a unique “chemokine signature” characterized by elevated serum GM-CSF and C-X-C motif chemokine ligand 10 (CXCL10)/Interferon gamma-induced protein 10 (IP-10) levels in a subset of COVID-19 patients who lost survival within 28 days of SARS-CoV-2 infections (Hue et al. 2020). Similarly, Blot M et al. reported a resembling elevation of plasma GM-CSF and CXCL10/IP-10 levels in some COVID-19 patients with worse outcomes (i.e., requirement for a longer duration of mechanical ventilation) (Blot et al. 2020). More recently, Thwaites, RS et al. discovered a progressive elevation of GM-CSF, CXCL10/IP-10 and IL-6 levels in some COVID-19 patients with severe thrombosis (Thwaites et al. 2021). Although IL-6 was equally elevated both in patients with COVID-19 or non-COVID-19 (e.g., influenza), only GM-CSF was prominently elevated in patients with severe COVID-19 (Thwaites et al. 2021), supporting the notion that GM-CSF serves as an important biomarker of COVID-19 disease severity (Hue et al. 2020; Gibellini et al. 2020; Blot et al. 2020; Thwaites et al. 2021; Zhao et al. 2021).

In response to severe SARS-CoV-2 infections, some hosts mount hyperactive inflammatory responses characterized by the excessive infiltration and activation of myeloid cells and consequent production of various cytokines and chemokines – the “cytokine storm” (Qiang et al. 2021). GM-CSF might be a key mediator of SARS-CoV-2-induced cytokine storm in a subset of COVID-19 patients with severe outcomes (Hamilton, 2020; Hue et al. 2020; Gibellini et al. 2020; Blot et al. 2020; Thwaites et al. 2021; Zhao et al. 2021). On the one hand, GM-CSF can promote myelopoiesis as well as recruitment of innate immune cells (e.g., monocytes, macrophages and dendritic cells) to SARS-CoV-2 infection sites (Hamilton, 2020; Qiang et al. 2021). On the other hand, GM-CSF can also polarize innate immune cells into pro-inflammatory phenotypes, promoting the production of proinflammatory cytokines (e.g., TNF, IL-1β and IL-6) and chemokines (e.g., MCP-1) (Hamilton, 2020).

Currently, a number of clinical trials are being planned to assess the efficacy of mAbs against GM-CSF (Clinical Trial Registry #: NCT04341116, NCT04351243, NCT04351152, NCT04376684) (Mehta et al. 2020; Lang et al. 2020; Hamilton et al. 2016) or GM-CSF receptor (Clinical Trial Registry #: NCT04399980, NCT04463004, and NCT04492514) (De et al. 2020). For instance, intravenous infusion of an anti-GM-CSF mAb (Lenzilumab, 600 mg, thrice) significantly reduced blood levels of IL-1α and IL-6 in 11 out of 12 patients with severe COVID-19 (Temesgen et al. 2020). Similarly, a neutralizing antibody against human GM-CSF receptor (mavrilimumab) significantly improved clinical outcome in 13 patients with severe COVID-19 pneumonia (De et al. 2020). In light of the emerging notion that GM-CSF might be a key biomarker of SARS-CoV-2-induced cytokine storm in a subset of COVID-19 patients with more severe outcome (Hue et al. 2020; Gibellini et al. 2020; Blot et al. 2020; Thwaites et al. 2021; Zhao et al. 2021), future GM-CSF-targeting clinical trials should be conducted in a subset of COVID-19 patients with particularly elevated blood GM-CSF levels.

## Conclusions

It is thus possible that SARS-CoV-2 spike protein-based vaccines may elicit protective antibodies capable of preventing SARS-CoV-2-elicited GM-CSF production and hyperactive “cytokine storm”. In light of the on-going efforts to develop effective SARS-CoV-2 vaccines, it may be important to assess the innate immune-modulating properties of all mRNA or adenoviral vaccine candidates and respective antibodies in experimental and clinical settings.

## Data Availability

The relevant supporting data is openly available in the website of the Journal of Leukocyte Biology: https://jlb.onlinelibrary.wiley.com/doi/10.1002/JLB.3COVCRA0920-628RR

## Abbreviations

ACE2: The angiotensin-converting enzyme 2
COVID-19: Coronavirus Disease 2019
CXCL10: C-X-C motif chemokine ligand 10
IL: Interleukin
IP-10: Interferon gamma-induced protein 10
GM-CSF: Granulocyte macrophage colony-stimulating factor
K_D_: Equilibrium binding constant
mAb: Monoclonal antibodies
MCP-1: Monocyte chemoattractant protein-1
MIP: Macrophage inflammatory protein
PBMCs: Human peripheral blood mononuclear cells
RBD: Receptor-binding domain
RBM: Receptor-binding motif
SPR: Surface plasma resonance
TNF: Tumor necrosis factor
